# Supplementary material for: Maternal Metal Ion Status Along Pregnancy and Perinatal Outcomes in a Group of Mexican Women
Source: Int J Mol Sci. 2024 Dec 8;25(23):13206. doi: 10.3390/ijms252313206 (PMC11642521; doi:10.3390/ijms252313206)
Supplement: Supplementary file 1 [file ijms-25-13206-s001.zip › Figure S1.pdf]

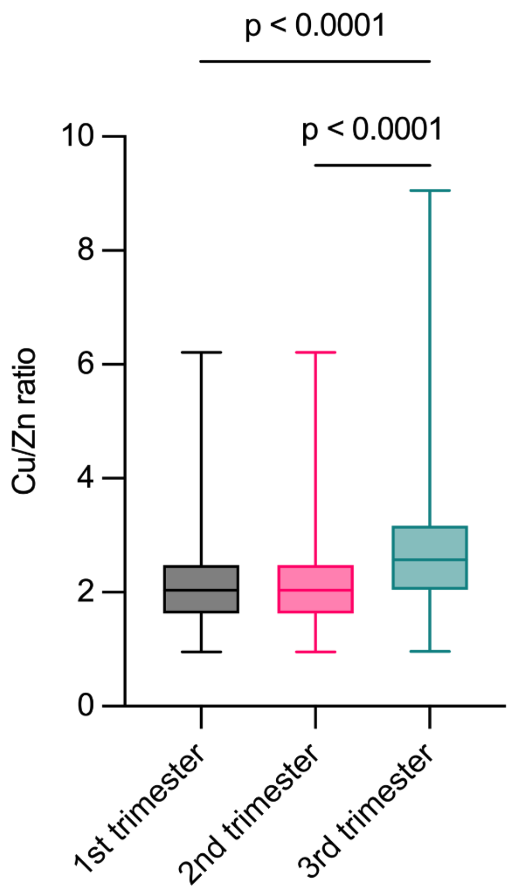


**Figure supplementary 1.** The Cu/Zn ratio increased significantly in the third trimester versus the first and second trimesters.
