# Supplementary material for: Maternal Metal Ion Status Along Pregnancy and Perinatal Outcomes in a Group of Mexican Women
Source: Int J Mol Sci. 2024 Dec 8;25(23):13206. doi: 10.3390/ijms252313206 (PMC11642521; doi:10.3390/ijms252313206)
Supplement: Supplementary file 1 [file ijms-25-13206-s001.zip › Figure S2.pdf]

Figure supplementary 2A

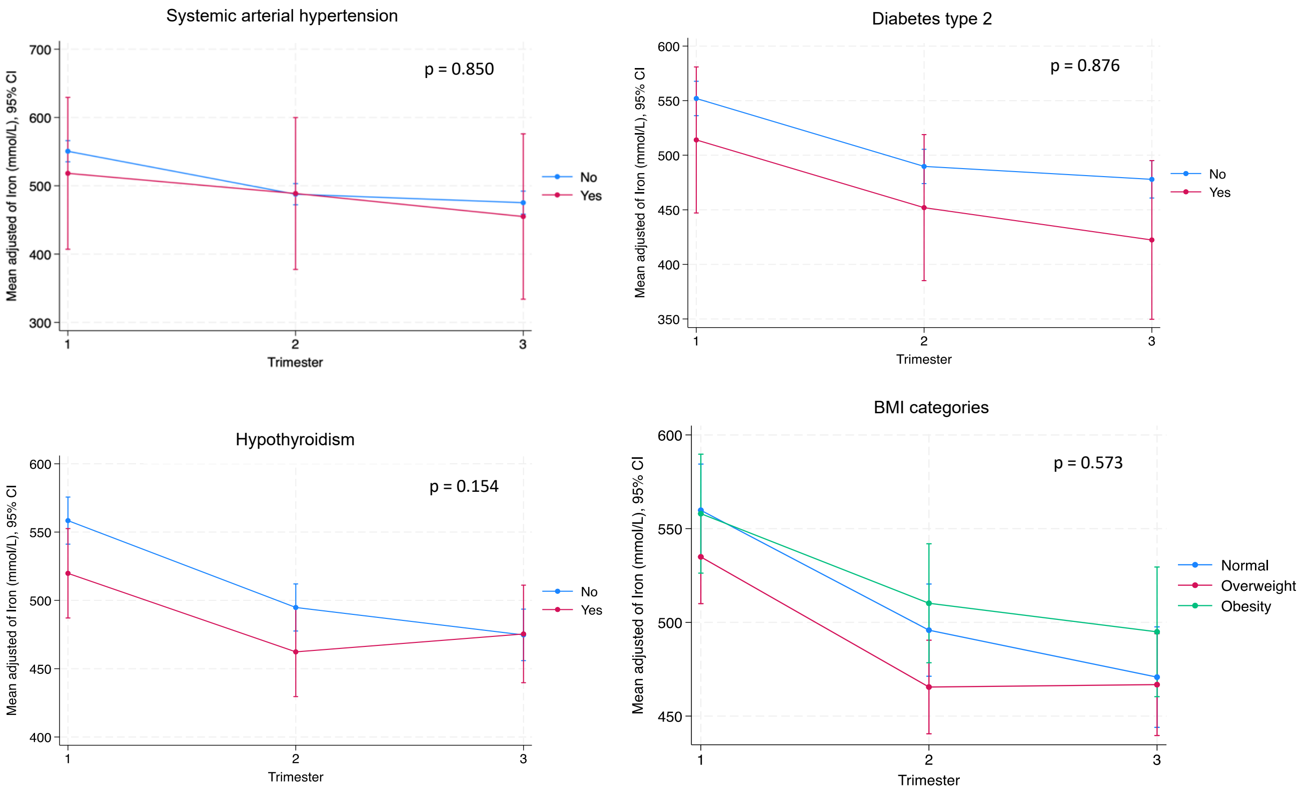

Results of the interaction effect between chronic degenerative disease diagnosed in pregnant women and iron levels in the three trimesters of pregnancy. No significant difference was reached in any of the cases.

Figure supplementary 2B

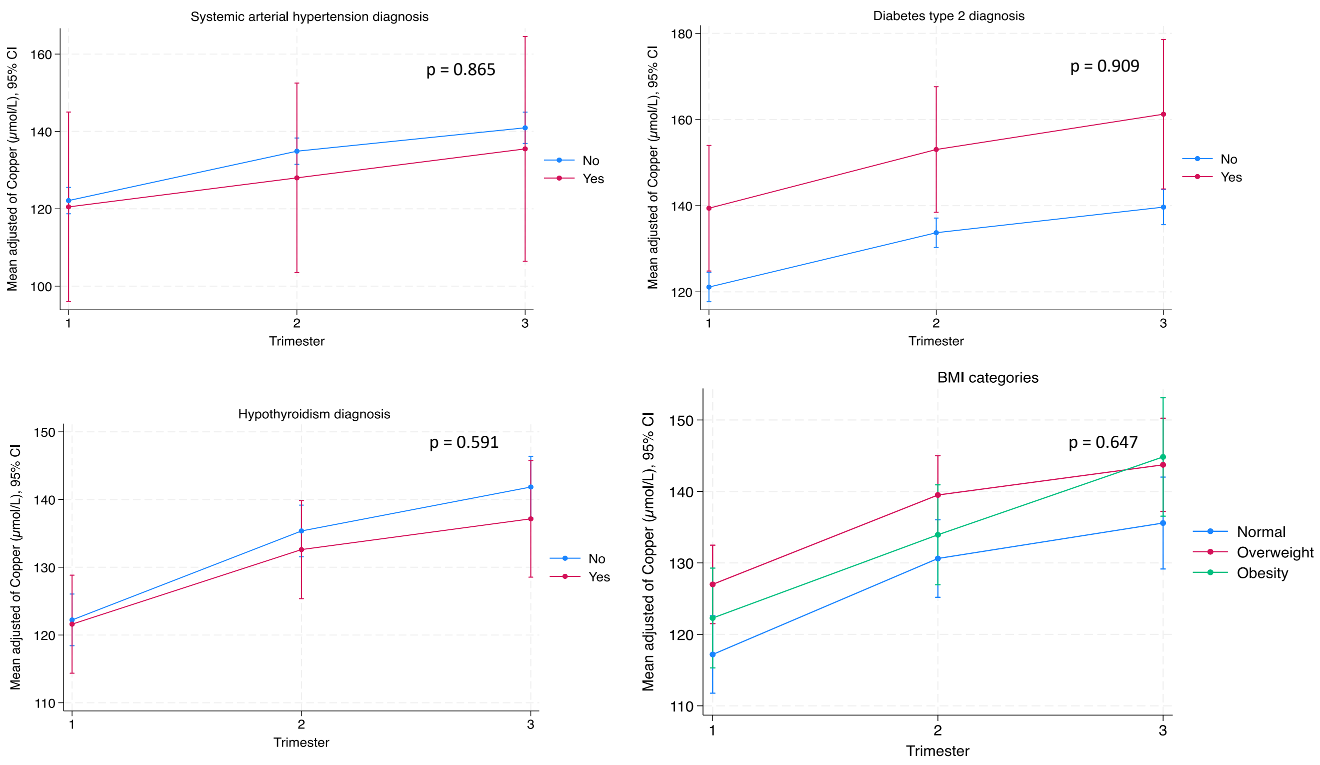

Results of the interaction effect between chronic degenerative disease diagnosed in pregnant women and copper levels in the three trimesters of pregnancy. No significant difference was reached in any of the cases.

Figure supplementary 2C

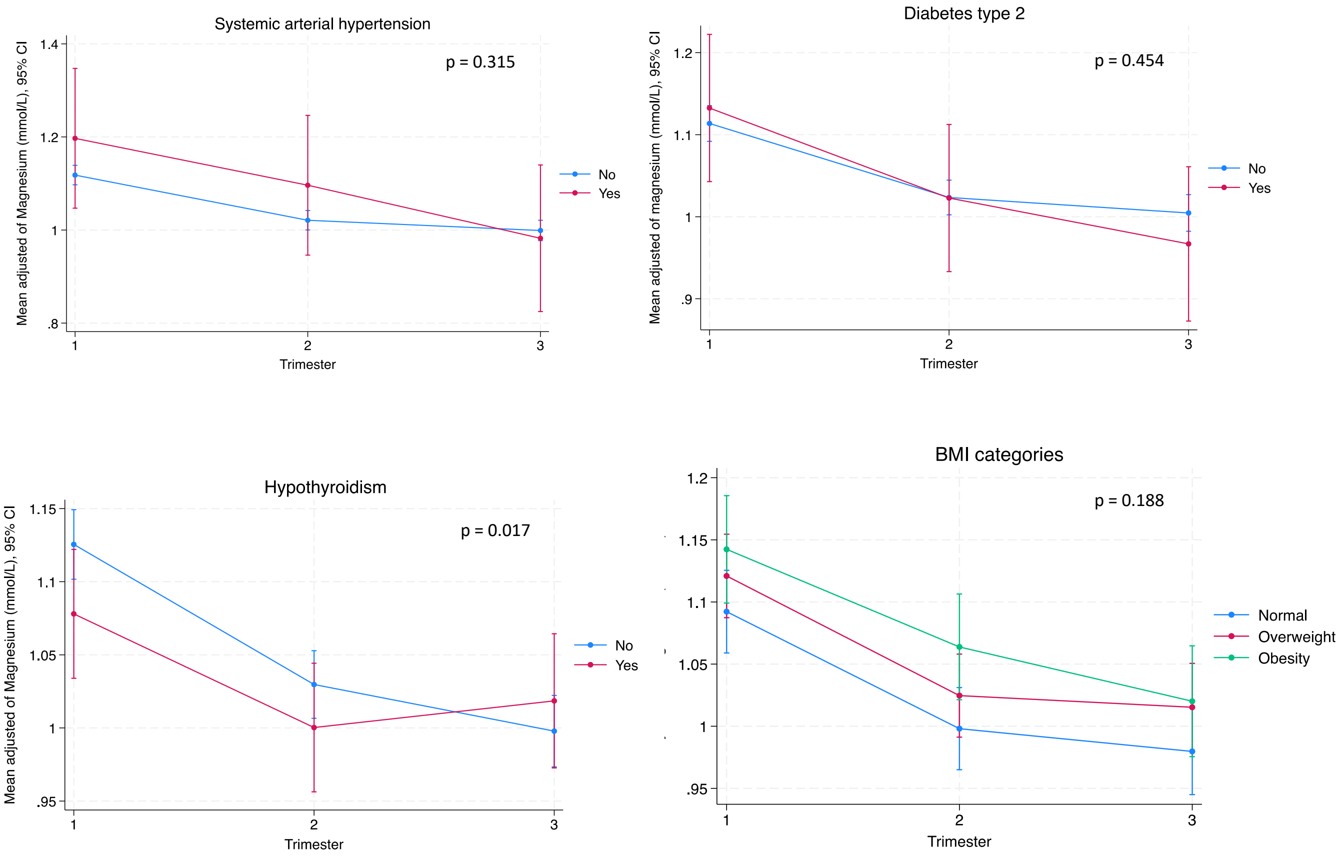

Results of the interaction effect between chronic degenerative disease diagnosed in pregnant women and magnesium levels in the three trimesters of pregnancy. No significant difference was reached in any of the cases.

Figure supplementary 2D

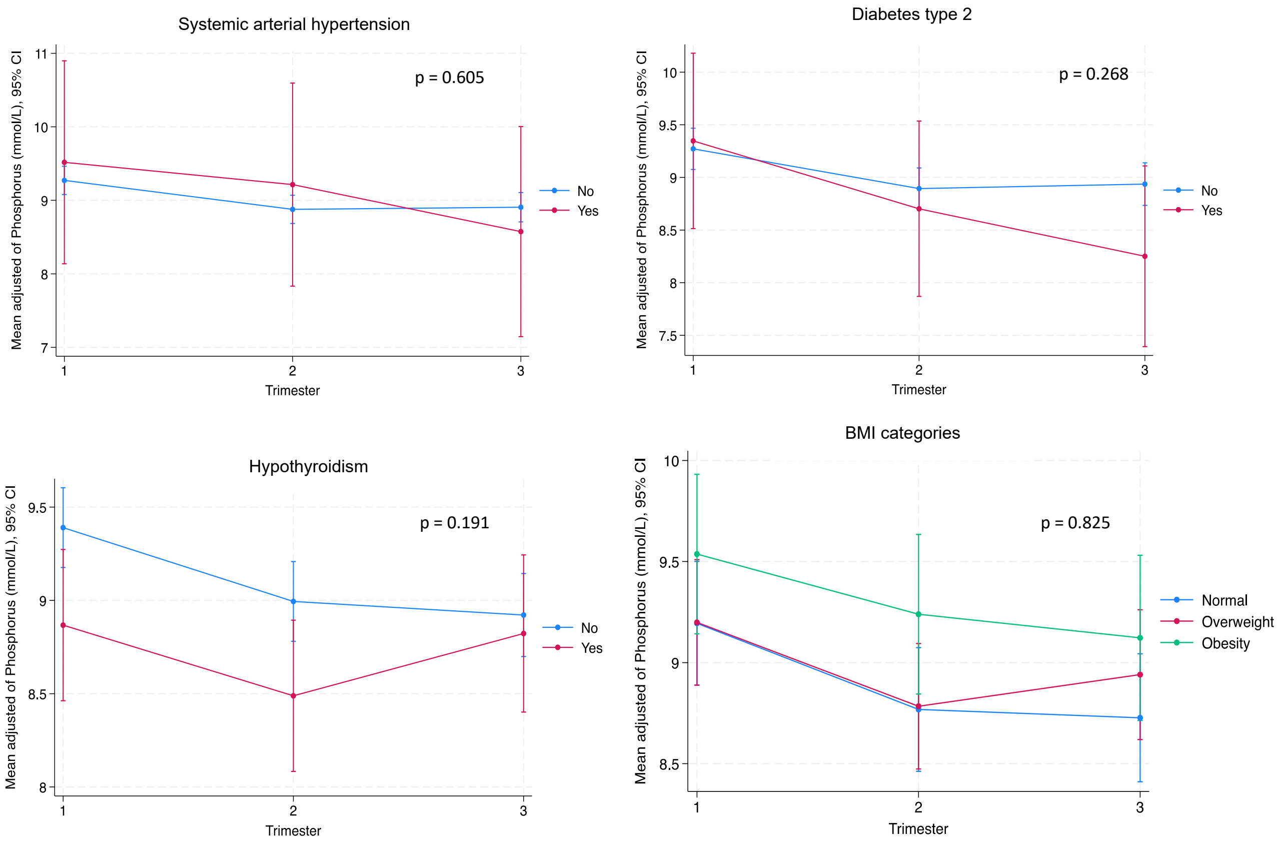

Results of the interaction effect between chronic degenerative disease diagnosed in pregnant women and phosphorus levels in the three trimesters of pregnancy. No significant difference was reached in any of the cases.

Figure supplementary 2E

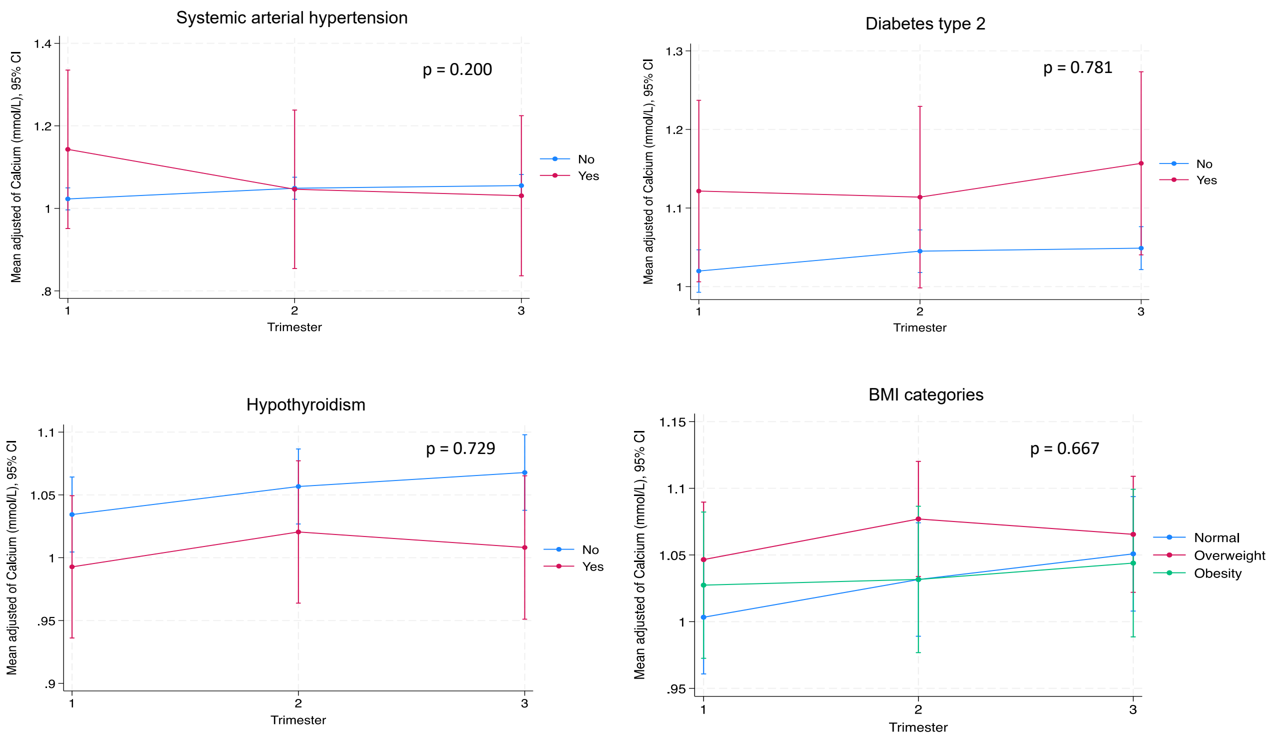

Results of the interaction effect between chronic degenerative disease diagnosed in pregnant women and calcium levels in the three trimesters of pregnancy. No significant difference was reached in any of the cases.

Figure supplementary 2F

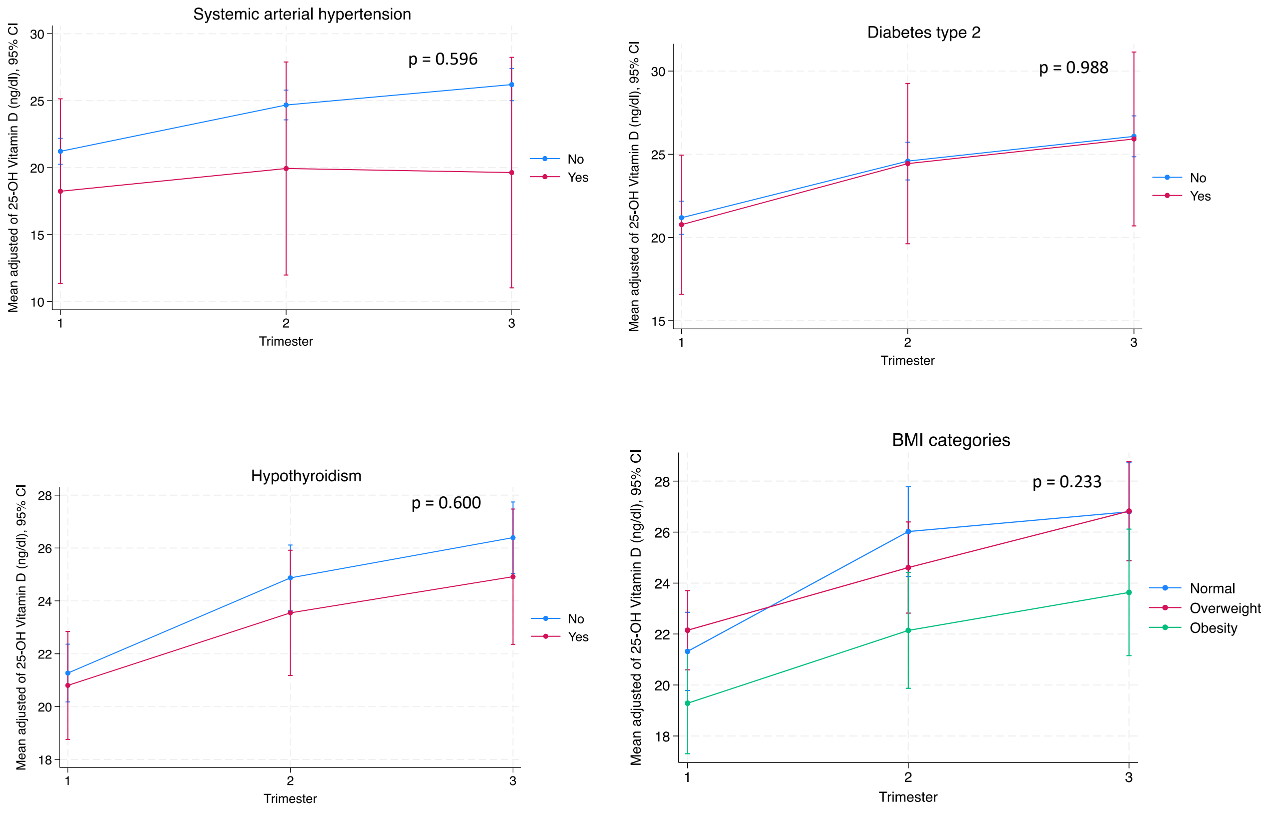

Results of the interaction effect between chronic degenerative disease diagnosed in pregnant women and vitamin D levels in the three trimesters of pregnancy. No significant difference was reached in any of the cases.
