# Supplementary material for: Maternal Metal Ion Status Along Pregnancy and Perinatal Outcomes in a Group of Mexican Women
Source: Int J Mol Sci. 2024 Dec 8;25(23):13206. doi: 10.3390/ijms252313206 (PMC11642521; doi:10.3390/ijms252313206)
Supplement: Supplementary file 1 [file ijms-25-13206-s001.zip › Figure S3.pdf]

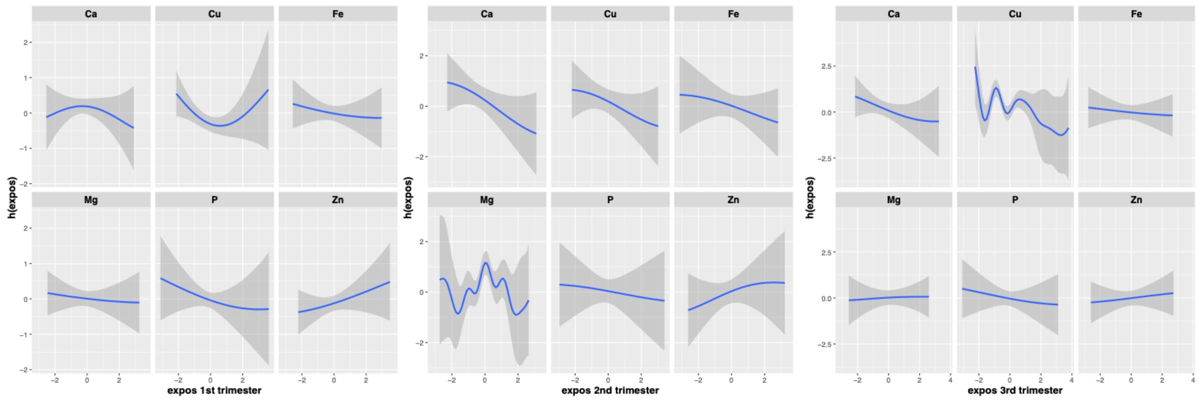

Figure supplementary 3A. Individual associations between metal ions and pregnant women who were diagnosed with preeclampsia. The plot suggests no linear effects of metal ions with patients diagnosed with this outcome across the three trimesters of pregnancy.

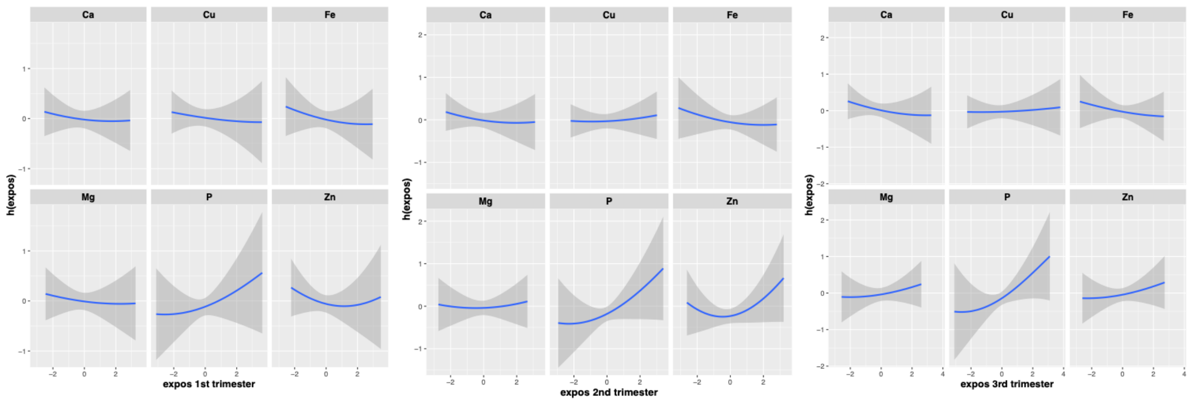

Figure supplementary 3B. Individual associations between metal ions and pregnant women who were diagnosed with gestational diabetes. The plot suggests no linear effects of metal ions with patients diagnosed with this outcome across the three trimesters of pregnancy.

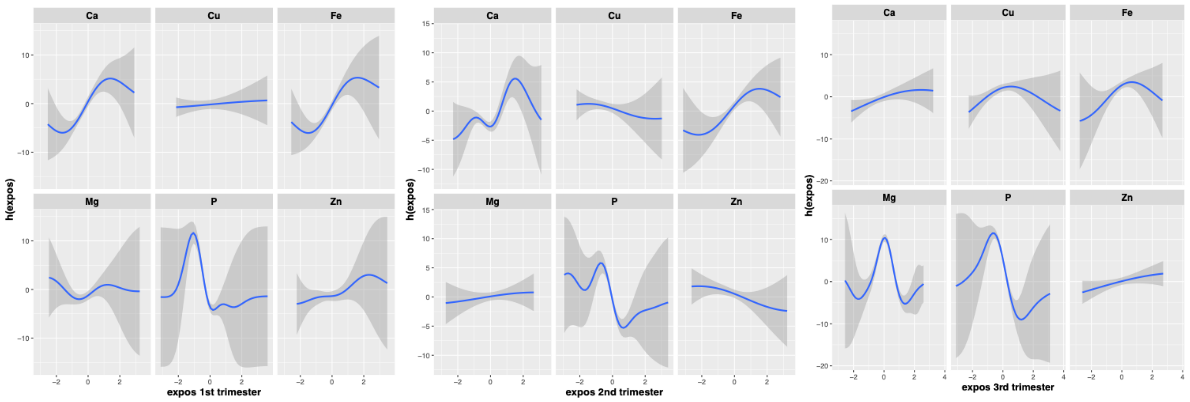

Figure supplementary 3C. Individual associations between metal ions and pregnant women who were diagnosed with obstetric hemorrhage during pregnancy. The plot suggests no linear effects of metal ions with patients diagnosed with this outcome across the three trimesters of pregnancy.

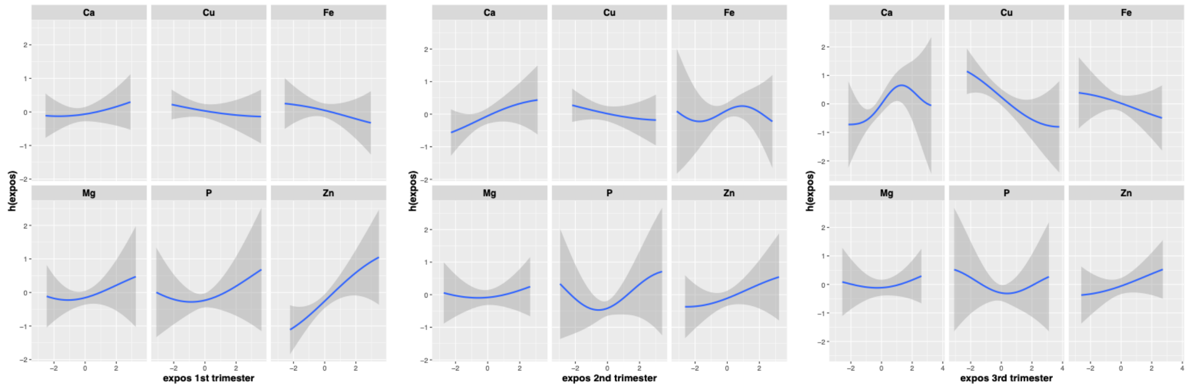

Figure supplementary 3D. Individual associations between metal ions and pregnant women whose newborns were admitted to the Intensive Care Unit. The plot suggests no linear effects of metal ions with patients diagnosed with this outcome across the three trimesters of pregnancy.

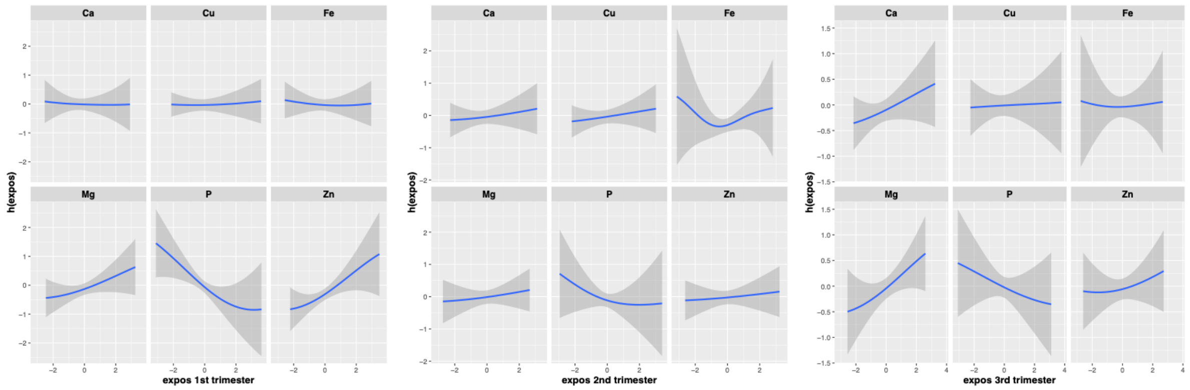

Figure supplementary 3E. Individual associations between metal ions and pregnant women whose newborns had low birth weight. The plot suggests no linear effects of metal ions with patients diagnosed with this outcome across the three trimesters of pregnancy.

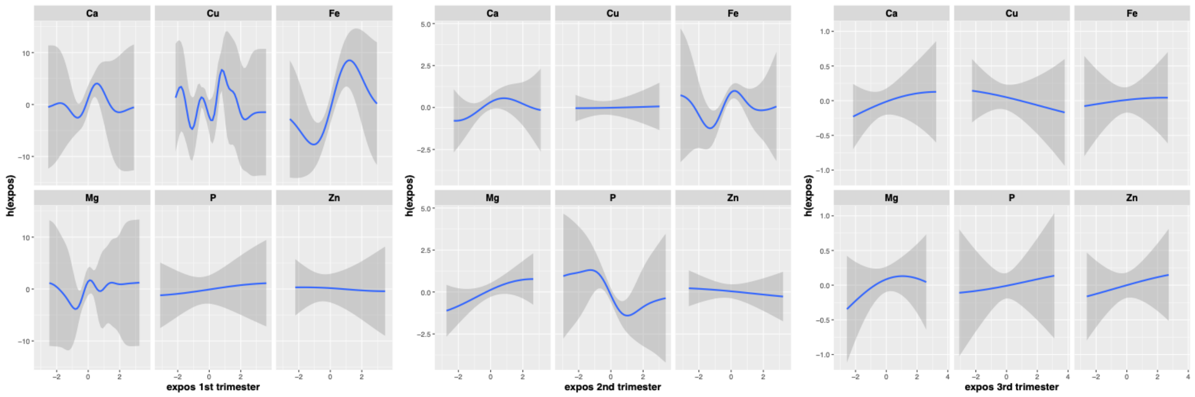

Figure supplementary 3F. Individual associations between metal ions and pregnant women whose delivered premature birth. The plot suggests no linear effects of metal ions with patients diagnosed with this outcome across the three trimesters of pregnancy.
