# Supplementary material for: Maternal Metal Ion Status Along Pregnancy and Perinatal Outcomes in a Group of Mexican Women
Source: Int J Mol Sci. 2024 Dec 8;25(23):13206. doi: 10.3390/ijms252313206 (PMC11642521; doi:10.3390/ijms252313206)
Supplement: Supplementary file 1 [file ijms-25-13206-s001.zip › Table S2.pdf]

Table supplementary 2

Estimates of coefficients in linear mixed model with interaction term

DESCRIPTION	T1			T2			T3			*p* for interaction
	ß	95% CI		ß	95% CI		ß	95% CI		
**Iron (mmol/L)**										
All[a]		Ref.		-61.06	-78.22	-43.89	-72.91	-78.22	-43.89	
Systemic arterial hypertension diagnosis[a]										
No		Ref.		-61.71	-79.05	-44.36	-73.11	-92.21	-54.01	0.850
Yes	-35.16	-147.46	77.14	-63.58	-175.84	48.67	-97.52	-219.52	24.47	
Diabetes type 2 diagnosis[a]										
No		Ref.		-61.27	-78.93	-43.-61	-72.13	-91.45	-52.80	0.876
Yes	-40.45	-109.39	28.48	-100.14	-169.19	-31.09	-129.76	-204.79	-54.72	
Hypothyroidism diagnosis[a]										
No		Ref.		-62.64	-81.73	-43.54	-81.81	-102.89	-60.74	0.154
Yes	-39.25	-76.38	-2.12	-96.44	-134.22	-58.66	-82.00	-122.30	-41.65	
BMI categories[b]										
Normal weight		Ref.		-61.91	-88.66	-35.17	-86.40	-115.32	-57.49	0.573
Overweight	-24.06	-59.19	11.08	-92.81	-128.48	-57.14	-90.05	-127.64	-52.47	
Obesity	-3.68	-43.95	36.59	-50.56	-90.94	-10.18	-65.03	-107.81	-22.24	
**Copper (µmol/L)**										
All[a]		Ref.		12.96	10.00	15.92	19.34	15.55	23.13	
Systemic arterial hypertension diagnosis[a]										
No		Ref.		13.09	10.10	16.08	19.45	15.62	23.28	0.865
Yes	-2.63	-27.14	21.87	5.23	-19.26	29.73	12.67	-16.42	41.76	
Diabetes type 2 diagnosis[a]										
No		Ref.		12.90	9.86	15.94	19.18	15.31	23.06	0.909
Yes	17.53	2.65	32.41	31.94	17.04	46.85	40.10	22.38	57.81	
Hypothyroidism diagnosis[a]										
No		Ref.		13.49	10.19	16.78	20.28	16.04	24.51	0.591
Yes	-0.80	-8.93	7.34	10.37	2.12	18.62	15.33	5.86	24.81	
BMI categories[b]										
Normal weight		Ref.		14.00	9.42	18.58	19.16	13.36	24.95	0.647
Overweight	10.07	2.40	17.73	22.72	14.96	30.48	27.44	18.85	36.03	
Obesity	4.46	-4.31	13.24	16.33	7.53	25.12	27.46	17.56	37.36	
**Zinc (µmol/L)**										
All[a]		Ref.		-5.16	-7.08	-3.24	-4.56	-6.61	-2.51	
Systemic arterial hypertension diagnosis[a]										
No		Ref.		-5.20	-7.14	-3.26	-4.55	-6.61	-2.48	0.915
Yes	-0.29	-13.33	12.75	-3.49	-16.52	9.54	-5.62	-19.19	7.95	

DESCRIPTION	T1<br>ß	95% CI		T2<br>ß	95% CI		T3<br>ß	95% CI		*p* for interaction
Diabetes type 2 diagnosis[a]										
No		Ref.		-5.09	-7.06	-3.12	-4.33	-6.42	-2.24	0.562
Yes	-1.90	-9.91	6.12	-8.37	-16.40	-0.34	-10.70	-19.06	-2.33	
Hypothyroidism diagnosis[a]										
No		Ref.		-5.59	-7.71	-3.47	-5.91	-8.18	-3.63	**0.033**
Yes	-8.22	-12.46	-3.98	-12.15	-16.46	-7.84	-8.25	-12.72	-3.78	
BMI categories[b]										
Normal weight		Ref.		-5.93	-8.92	-2.94	-5.32	-8.45	-2.19	0.963
Overweight	-2.71	-6.78	1.37	-7.51	-11.64	-3.37	-6.71	-10.98	-2.45	
Obesity	-0.42	-5.09	4.25	-4.94	-9.62	-0.25	-4.68	-9.51	0.15	
**Magnesium (mmol/L)**										
All[a]		Ref.		-0.10	-0.11	-0.07	-0.11	-0.13	-0.09	
Systemic arterial hypertension diagnosis[a]										
No		Ref.		-0.09	-0.11	-0.07	-0.11	-0.13	-0.09	0.315
Yes	0.07	-0.08	0.22	-0.02	-0.17	0.13	-0.14	-0.30	0.02	
Diabetes type 2 diagnosis[a]										
No		Ref.		-0.09	-0.11	-0.07	-0.11	-0.13	-0.09	0.454
Yes	0.02	-0.07	0.11	-0.09	-0.18	0.002	-0.15	-0.24	-0.05	
Hypothyroidism diagnosis[a]										
No		Ref.		-0.10	-0.12	-0.07	-0.13	-0.15	-0.10	**0.017**
Yes	-0.05	-0.10	0.002	-0.13	-0.18	-0.07	-0.11	-0.16	-0.05	
BMI categories[b]										
Normal weight		Ref.		-0.09	-0.13	-0.06	-0.11	-0.15	-0.08	0.788
Overweight	0.03	-0.02	0.08	-0.07	-0.12	-0.02	-0.08	-0.13	-0.03	
Obesity	0.05	0.004	0.10	-0.03	-0.08	0.03	-0.07	-0.13	-0.02	
**Phosphorus (mmol/L)**										
All[a]		Ref.		-0.34	-0.55	-0.12	-0.29	-0.52	-0.06	
Systemic arterial hypertension diagnosis[a]										
No		Ref.		-0.33	-0.55	-0.12	-0.28	-0.50	-0.05	0.605
Yes	0.17	-1.20	1.56	-0.09	-1.47	1.29	-0.74	-2.16	0.69	
Diabetes type 2 diagnosis[a]										
No		Ref.		-0.32	-0.54	-0.10	-0.25	-0.48	-0.02	0.268
Yes	0.05	-0.80	0.90	-0.52	-1.38	0.32	-0.95	-1.83	-0.08	
Hypothyroidism diagnosis[a]										
No		Ref.		-0.34	-0.58	-0.10	-0.38	-0.64	-0.13	0.191
Yes	-0.54	-1.00	-0.09	-0.88	-1.35	-0.42	-0.52	-0.99	-0.04	
BMI categories[b]										
Normal weight		Ref.		-0.34	-0.68	-0.01	-0.37	-0.72	-0.02	0.825

DESCRIPTION	T1 ß	T1 95% CI		T2 ß	T2 95% CI		T3 ß	T3 95% CI		*p* for interaction
Overweight	0.02	-0.41	0.45	-0.36	-0.79	0.08	-0.15	-0.60	0.30	
Obesity	0.30	-0.20	0.79	0.05	-0.45	0.54	-0.04	-0.55	-0.47	
**Calcium (µmol/L)**										
All[a]		Ref.		0.02	-0.001	0.05	0.03	0.01	0.06	
Systemic arterial hypertension diagnosis[a]										
No		Ref.		0.03	0.001	0.05	0.04	0.01	0.06	0.200
Yes	0.11	-0.08	0.30	0.02	-0.17	0.21	0.002	-0.19	0.20	
Diabetes type 2 diagnosis[a]										
No		Ref.		0.03	0.001	0.05	0.03	0.01	0.06	0.781
Yes	0.10	-0.02	0.22	0.09	-0.03	0.21	0.13	0.01	0.25	
Hypothyroidism diagnosis[a]										
No		Ref.		0.02	-0.004	0.05	0.04	0.01	0.06	0.729
Yes	-0.04	-0.11	0.02	-0.02	-0.08	0.05	-0.02	-0.09	0.04	
BMI categories[b]										
Normal weight		Ref.		0.03	-0.01	0.07	0.05	0.01	0.09	0.667
Overweight	0.05	-0.01	0.11	0.07	0.01	0.14	0.07	0.01	0.13	
Obesity	0.02	-0.05	0.09	0.02	-0.05	0.09	0.04	-0.03	0.11	
**25-OH Vitamin D, (ng/dl)**										
All[a]		Ref.		3.42	2.50	4.33	4.91	3.88	5.93	
Systemic arterial hypertension diagnosis[a]										
No		Ref.		3.46	2.53	4.38	4.98	3.94	6.01	0.596
Yes	-2.98	-9.95	3.98	-1.28	-9.30	6.73	-1.59	-10.25	7.07	
Diabetes type 2 diagnosis[a]										
No		Ref.		3.40	2.46	4.35	4.89	3.84	5.94	0.988
Yes	-0.42	-4.73	3.89	3.25	-1.68	8.18	4.73	-0.60	10.06	
Hypothyroidism diagnosis[a]										
No		Ref.		3.60	2.58	4.63	5.12	3.97	6.28	0.600
Yes	-0.47	-2.78	1.85	2.28	-0.35	4.90	3.65	0.85	6.44	
BMI categories[b]										
Normal weight		Ref.		4.70	3.28	6.12	5.48	3.86	7.09	0.233
Overweight	0.83	-1.342	3.01	3.29	0.92	5.66	5.50	3.01	7.99	
Obesity	-2.03	-4.52	0.46	0.82	-1.92	3.57	2.31	-0.61	5.23	

[a] Models adjusted by age(years), glucose, triglycerides (mg/dl), cholesterol (mg/dl), 25-OH vitamin D (ng/dl), body mass index, multivitamin supplementation (yes/no) and socioeconomic status (low/medium-high).); [b]Models adjusted by age(years), glucose, triglycerides (mg/dl), cholesterol (mg/dl), 25-OH vitamin D (ng/dl), body mass index, and multivitamin supplementation (yes/no); Abbreviations: OR: Odds ratio; CI: Confidence Intervale; T1: Trimester 1; T2: Trimester 2; T3: Trimester 3; Ref: Reference category.
